# A Botanical and Medical Account of the Quassia Simaruba, or Tree Which Produces the Cortex Simaruba

**Published:** 1790

**Authors:** William Wright

**Affiliations:** Physician General in Jamaica


X. *A botanical and medical Account of the Quaffia Simaruba, or Tree which produces the Cortex Simaruba. By* William Wright, *M. D. F. R. S. Lond. and Edin. and Phyfician General in Jamaica.—From the Tranfactions of the Royal Society of Edinburgh*, Vol. II.

*An hiftorical Account of the* SIMARUBA BARK.

THE firft knowledge we had of the cortex fimarubæ was in the year 1713. Some of it was fent to France to M. le Comte de Porchartrain, the Secretary of State, as the

 bark bark of a tree, called by the natives Simarouba, which they employed with good ſucceſs in dyſentery.

In 1741, M. Geoffroy, in ſpeaking of this bark, ſays, " Eſt cortex radicis arboris ignotæ, " in Guiana naſcentis, et ab incolis ſimarouba " nuncupatæ : coloris eſt ex albo flaveſcentis, " nullo odore præditus, ſaporis ſubamari, len- " tiſcentibus fibris conſtans, candido, leviſſimo, " inſipidoque, radicum, ſtipitum, truncique " ligno hærens, a quo facile ſeparatur."

In 1753 and 1760, Linnæus makes the ſimaruba to be a ſpecies of piſtacia, or the terebinthinus major, betulæ cortice, fructu triangulari of Sloan. Jam. 289. t. 99.

In 1756, Dr. Patrick Browne publiſhed his Civil and Natural Hiſtory of Jamaica. At page 345 he deſcribes the terebinthinus, or birch and turpentine tree. The bark of the roots, ſays he, is thought to be the ſimarouba of the ſhops.

In 1763, Linnæus makes ſimaruba to be the burſera gummifera, and refers to the piſtacia of former editions of the Species Plantarum; and to Sloan and Browne, as above cited. In the appendix a reference is made to the terebinthinus binthinus Americana polyphylla. Commelin. Hort. 1. p. 149, and to Catefby's gum elemi tree.

M. Jacquin vifited all the Weft-India iflands, and made many difcoveries of new plants. He examined the roots of the burfera gummifera, and found its bark very different from the fimaruba bark.

In 1772 I employed all my fpare hours in examining the plants of Jamaica. In this delightful walk of fcience I difcovered and afcertained many hundreds of new plants which had efcaped the diligence of former botanifts; amongft others, the tree which produces the fimaruba bark.

In 1773 fpecimens of the fructification were fent in fpirits, accompanied with a botanical account of the tree, to my late worthy friend Dr. Hope, Profeffor of Botany in the Univerfity of Edinburgh; alfo fome dried bark from the roots. The following year fpecimens, with fimilar defcription, were tranfmitted to my late learned and valuable friend Dr. John Fothergill, of London, who fent them to the celebrated Linnæus at Upfal, as appears by Profeffor Murray's Apparatus Medicaminum, Vol. III.

page 458 *, article Simaruba. Dr. Fothergill caufed elegant drawings to be made of this plant; and thefe drawings I now have the honour of prefenting to the Royal Society of Edinburgh.

It is here proper to remark, that this paper was read before the Philofophical Society of this place, and committed for publication in 1778. At the time when that Society obtained the Royal Charter I chanced to be abroad. On my return to Edinburgh I withdrew the communication, to correct and add to my account of this important article of materia medica.

### *Defcription of the Tree.*

The tree now to be defcribed is common in all the woodlands in Jamaica. It grows to a great height and confiderable thicknefs. The trunks of the old trees are black and a little

* Qualis vera ejufdem arbor fit, jamjam Aubletii indagine cognofcimus; ut tamen et mihi monere incumbat, b. Linnæum equitem, litteris jam anno 1776, ineunte mihi datis, antequam Aubletii elegantiffimum opus illi innotefceret, fignificaffe, Simarubam Quaffiæ fpecies a fe haberi. Ille autem fimarubæ cortex, quo Cl. Wright, arborem in Jamaica vulgarem veftitam effe innuit, pariter in alvi profluviis efficaci, &c.

furrowed:

furrowed: thofe of the young trees fmooth and gray, with here and there a broad yellow fpot.

The infide bark of the trunk and branches is white, fibrous, and tough. It taftes flightly bitter. On cutting or ftripping off this bark, no milky juice iffues, as has been mentioned by various authors.

The wood is hard, and ufeful for buildings. It fplits freely, and makes excellent ftaves for fugar hogfheads. It has no fenfible bitter tafte.

The branches are alternate and fpreading.

The leaves are numerous and alternate. On the upper fide they are fmooth, fhining, and of a deep green colour; on the under fide they are white.

The flowers appear about the beginning of April. They are of a yellow colour, and placed on fpikes beautifully branched.

The fruit is of that kind called a drupa, and is ripe towards the end of May. It is cf an oval fhape; is black, fmooth, and fhining. The pulp is flefhy and foft; the tafte a naufeous fweet. The nut is flattened, and on one fide winged. The kernel is fmall, flat, and taftes fweet.

The natural number of thefe drupæ is five on each common receptacle; but, for the moft part, part, there are only two or three; the reſt abort by various accidents.

The roots are thick, and run ſuperficially under the ſurface of the ground to a conſiderable diſtance. The bark is rough, ſcaly, and warted. The inſide, when freſh, is a full yellow, but when dry paler. It has but little ſmell. The taſte is bitter, but not very diſagreeable. This is the true Cortex Simarubæ of the ſhops.

This tree is known in Jamaica by the names of Mountain Damſon, Bitter Damſon, and Stave Wood. The ſhops are ſupplied with this bark from Guiana; but now we may have it from our own iſlands at a moderate expence.

On examining the fructification, I found this tree to be a ſpecies of Quaſſia. Under that name I ſent it to Europe, and Linnæus adopted it into his ſyſtem.

There are male flowers on one tree, and female flowers on another; and this is invariably the caſe in Jamaica.

*Senſible Qualities of the Cortex Simarubæ.*

I can diſcover no aſtringency in the cortex ſimarubæ, either by the taſte, or by the various teſts to which I ſubjected it; nor is there any mucilaginous quality to be perceived in the recent cent bark, or in the decoction of that which has been dried.

*Its medical Virtues in general.*

Moſt authors who have written on the ſimaruba agree, that in fluxes it reſtores the loſt tone of the inteſtines and allays their ſpaſmodic motions, promotes the ſecretions by urine and perſpiration, removes the lowneſs of ſpirits attendant on dyſenteries, and diſpoſes the patient to ſleep; the gripes and teneſmus being, by the uſe of it, taken off, and the ſtools changed to their natural colour and conſiſtence.

In a moderate doſe it occaſions no diſturbance or uneaſineſs; but in large doſes it produces ſickneſs at ſtomach and vomiting. Negroes are leſs affected by it than white people.

*Preparation of Simaruba Bark.*

The ſimaruba bark yields its qualities to water, either in cold infuſion or in decoction. I prefer the latter. Phyſicians have preſcribed the bark in different quantities; but it ſeems now agreed that the following proportion is the beſt:

Two drachms of ſimaruba bark, boiled from twenty-four ounces of water to twelve ounces; then ſtrained.

This is to be divided into three equal parts, and the whole taken in twenty-four hours.

When the ſtomach is reconciled to it, three drachms may be boiled in the ſame quantity of water, and taken as above mentioned. Some join aromatics to the decoction of this bark; others give a few drops of laudanum with each doſe. The decoction is to be drank daily till the diſorder is cured, which ſometimes happens in a few days, and at other times it may require weeks to perfect a cure.

*Of the Effects of Simaruba in particular Diſeaſes.*

Having thus treated of the ſimaruba in general, I am now to mention its uſe and effects more particularly in different diſeaſes, and firſt in the dyſentery. In the years 1718 and 1723 an epidemic flux prevailed in France, and ſwept off a great number of people of all ages and of both ſexes. This diſorder not only reſiſted all the medicines given, but was aggravated by ſmall doſes of ipecacuanha, the mildeſt purgatives, and all aſtringents. The diſorder was happily cured by the ſimaruba.

M. Juſſieu

M. Juffieu ufed this bark for fifteen years in obftinate dyfenteries with great fuccefs; and continued its exhibition, although the catamenia in women, or hæmorrhage from piles in men, occurred during the cure.

Modern phyficians have found, from experience, that this medicine is only fuccefsful in the third ftage of dyfentery, where there is no fever, where too the ftomach is no way hurt, and where the gripes and tenefmus are only continued by a weaknefs of the bowels. In fuch cafes Dr. D. Monro gave two or three ounces of the decoction every five or fix hours, with four or five drops of laudanum, and found it a very ufeful remedy.

The late Sir John Pringle, Dr. Huck Saunders, and many others, prefcribed the cortex fimarubæ in old and obftinate dyfenteries and diarrhœas, efpecially thofe brought from warm climates. Fluxes of this fort, which were brought home from the fieges of Martinico and the Havannah, were completely and fpeedily cured by this bark. The urine which, in thofe cafes, had been high coloured and fcanty, was now voided in great abundance, and perfpiration reftored. Dr. James Lind, at Haflar Hofpital, fays, that the fimaruba produced thefe effects fooner, and more certainly, when given in fuch quantity as to naufeate the ftomach. Dr. Huck Saunders remarks, that if the fimaruba did not give relief in three days, he expected little benefit from its farther ufe; but others have found it efficacious in fluxes, after a continued ufe for feveral weeks. Authors have cautioned us againft the ufe of this bark where the inteftines are ulcerated and difpofed to cancer after fluxes.

In diarrhœas, from abforption of pus, the fimaruba has given relief; the former difcharge from fuch ulcers having been reftored, and the pus meliorated.

Lienteria itfelf, and even hepatic fluxes, have been cured by the fimaruba, after other medicines were tried without fuccefs. *Vide* Act. Natur. curiof. Tom. II. p. 80—82.

In putrid fevers, as we are told, attended with coldnefs of the extremities, colliquative fweats and ftools, and great dejection of fpirits, this bark performed wonders, and many recovered by its ufe. *Vide* Roupe de Morbis Navigantium, p. 311.

Habitual colics, with bloody ftools, attended with fever and delirium, have been radically cured by the fimaruba bark.

Immoderate

Immoderate fluxes of the menſes and from piles have been happily ſtopped by this medicine; and it would appear, from ſome late trials, that fluor albus has been remedied by the ſame bark.

De Haen found the ſimaruba to be an excellent vermifuge; and uſed it with ſucceſs in diſeaſes depending on worms, particularly in fluxes.

My own experience, and that of many living friends, are convincing proofs to me of the efficacy of this medicine; and I hope the ſimaruba bark will ſoon be in more general uſe.

### QUASSIA SIMARUBA*.

### FLOS MASCULUS.

*Cal.* Perianthium monophyllum, parvum, quinquefidum, denticulis ovatis, erectis.

*Cor.* Petala quinque, ſeſſilia, æqualia, lanceolata, ſubrevoluta, calyce triplo longiora, calyci inſerta. *Nectarium* ex ſquamis decem, ovatis, villoſis, baſi filamentorum interiori inſertis.

* To this botanical deſcription the Society have annexed two excellent engravings of the *Quaſſia Simaruba mas* and *Quaſſia Simaruba feminea*, from the drawings preſented by the Author; but for theſe we muſt refer our readers to the work itſelf.—EDITOR.

*Stam.* Filamenta decem, filiformia, æqualia, longitudine corollæ. *Antheræ* oblongæ, incumbentes; in centro floris corpus carnosum, orbiculatum, decem-sulcatum.

*Pistillum* nullum.

FLOS FEMINEUS.

*Calyx* et *Corolla*, ut in flore masculo.

*Pistillum.* Germina quinque subrotunda, introrsum coalita. *Stylus* cylindraceus, erectus, quinque-partitus, longitudine corollæ. *Stigmata* subulata, recurvata, persistentia.

*Pericarpium.* Drupæ quinque laterales, distantes, receptaculo orbiculato, carnoso insertæ.

*Semina.* Nux oblongo-ovata, acuminata, unilocularis. *Nucleus* compressus.

INFLORESCENTIA.

Panicula composita. Pedicellis subjicitur stipula lanceolata, petiolata. Folia alternato-pinnata. Foliola oblonga, obtusa, nitida, integra, basi attenuata, subsessilia; costis lateralibus nervosis.

